# Supplementary material for: Rearing in an Enriched Environment Ameliorates the ADHD-like Behaviors of Lister Hooded Rats While Suppressing Neuronal Activities in the Medial Prefrontal Cortex
Source: Cells. 2022 Nov 17;11(22):3649. doi: 10.3390/cells11223649 (PMC9688563; doi:10.3390/cells11223649)
Supplement: Supplementary file 1 [file cells-11-03649-s001.zip › Supplementary Figures.pdf]

Supplementary Figures

Rearing in an enriched environment ameliorates the ADHD-like behaviors of Lister hooded rats while suppressing neuronal activities in the medial prefrontal cortex

Ryo Utsunomiya[1], Kanta Mikami, Tomomi Doi, Mohammed E. Choudhury, Toshihiro Jogamoto[2], Naohito Tokunaga[3], Eiichi Ishii[4], Mariko Eguchi[2], Hajime Yano[1], Junya Tanaka[1*]

[1] Department of Molecular and Cellular Physiology, Graduate School of Medicine, Ehime University, Toon 791-0295, Ehime, Japan
[2] Department of Pediatrics, Graduate School of Medicine, Ehime University, Toon 791-0295, Ehime, Japan
[3] Division of Medical Research Support, the Advanced Research Support Center (ADRES), Ehime University, Toon 791-0295, Ehime, Japan
[4] Department of Pediatrics, Imabari City Hospital, Imabari 794-0026, Ehime, Japan
* Correspondence: jtanaka@m.ehime-u.ac.jp; Tel.: +81899605241

Supplementary Figure S1

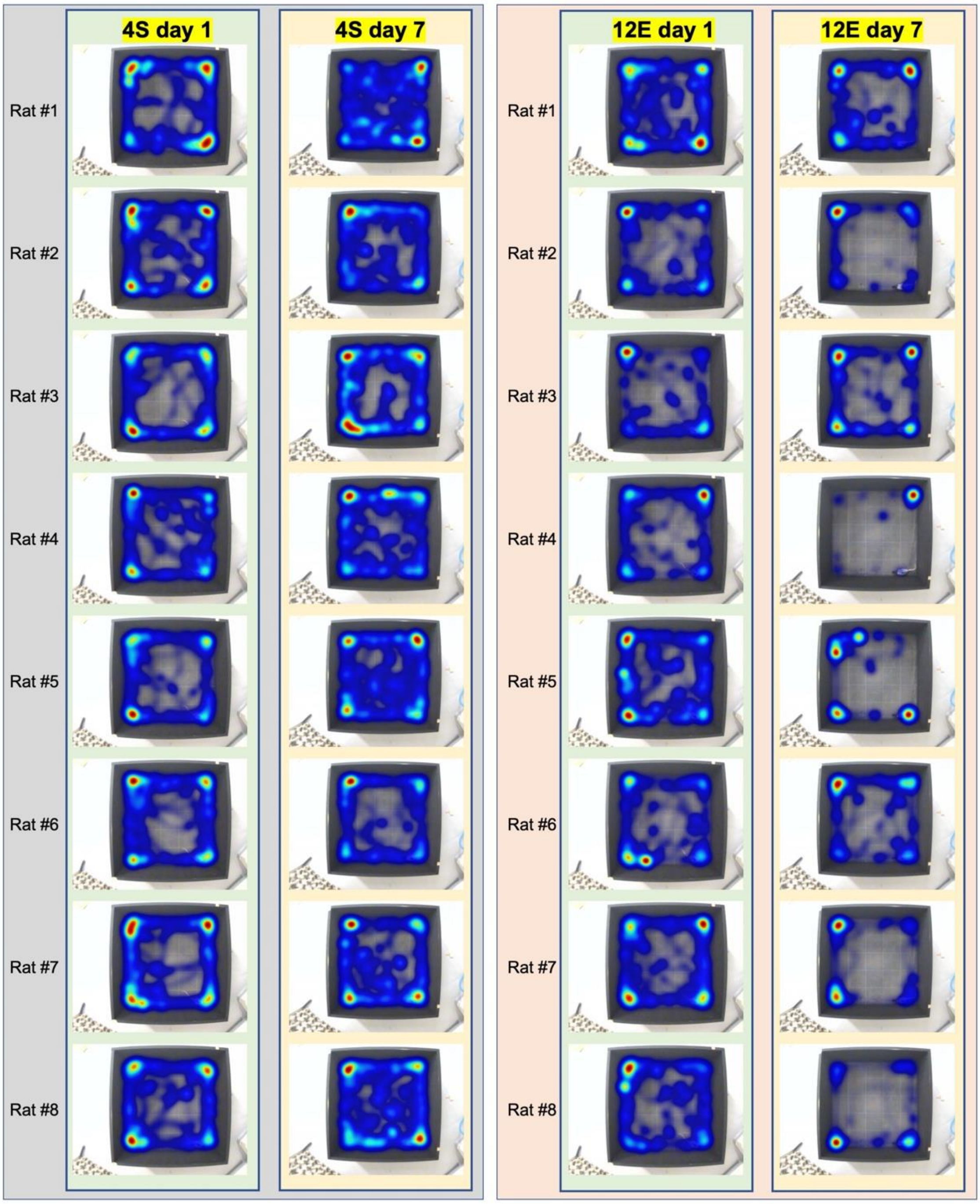

All heatmap data used for statistical analysis for Figure 2C.

Supplementary Figure S2

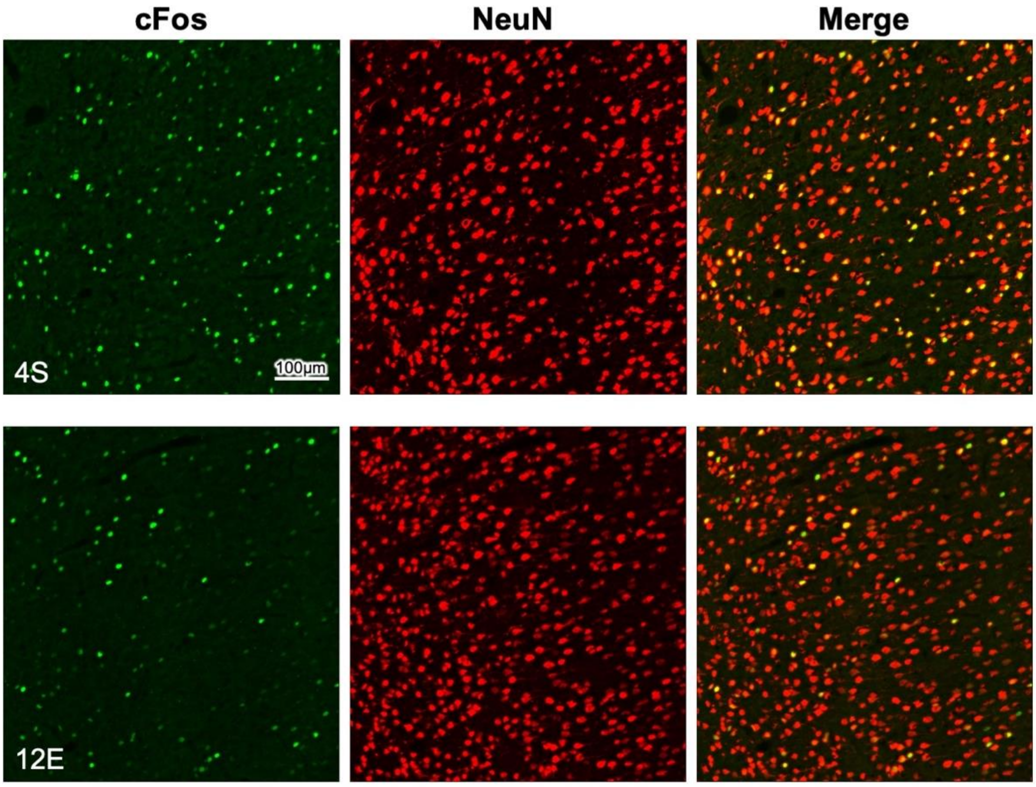

Enlarged micrographs displaying distribution of $cFos^+/NeuN^+$ neurons in the PrL of 4S and 12E LHRs.
